# Effects of different interventions on internet addiction: a systematic review and network meta-analysis

**DOI:** 10.1186/s12888-023-05400-9

**Published:** 2023-12-08

**Authors:** Yuqiong Zhu, Haihan Chen, Junda Li, Xian Mei, Wenjuan Wang

**Affiliations:** 1 School of Mental Health, Bengbu Medical College, Bengbu, Anhui 233030 China; 2 School of Second Clinical College, Zhejiang Chinese Medical University, Hangzhou, Zhejiang 310053 China; 3 School of Qian Xuesen College, Xi’an Jiaotong University, Xi’an, Shanxi 710049 China

**Keywords:** Internet addiction, Intervention, Network meta-analysis

## Abstract

**Background:**

Globally, Internet is a recognized form of leisure, but there are growing apprehensions about the increasing number of individuals developing an addiction to it. Recent research has focused on social issues associated with internet addiction (IA). However, the treatment of IA is currently unclear. This study aimed to explore the relationship between IA treatment outcomes and different intervention strategies through systematic review and data analysis of patients who received different intervention modes.

**Methods:**

A meta-analysis was conducted using RevMan 5.4 and Stata 14.2 on 57 literature research data from five Chinese and English databases, PubMed, Embase, Web of Science, Wanfang and CNKI.

**Result:**

A total of 57 randomized controlled trials (RCTs) were included in this network meta-analysis involving 3538 IA patients and 13 different interventions. The network meta-analysis results demonstrated that the top four interventions were: rTMS + CBT, drug + others, rTMS, and electro-acupuncture + CBT.

**Conclusion:**

Our study indicated that comprehensive therapy had an optimal therapeutic effect on IA patients and rTMS + CBT ranked first among all therapeutic indicators of intervention, indicating optimal clinical effectiveness.

## Background

The internet has revolutionized communication, work, and access to information, becoming an integral part of modern life with numerous benefits and conveniences for users. However, this ubiquitous technology also has a darker side. Concerns about excessive Internet use have been raised in recent years, leading to the concept of Internet addiction (IA). According to statistics, the incidence of IA among Chinese college students is 11% [1]. IA was first proposed by psychologist Goldberg I. In 1996, Young confirmed that IA should be a true clinical psychological disorder [2]. IA was defined as the uncontrolled behavior of accessing the internet without substance, manifested as significant social and psychological impairment of individuals due to excessive use of the internet [3]. Although IA has not yet been formally incorporated into the framework of psychopathology, it is a potential problem both in terms of prevalence and public awareness, with many similarities to existing recognized barriers. Some studies have shown that IA may be related to abnormal activity in multiple brain regions and neurotransmitter systems, such as the prefrontal cortex, amygdala, ventral prefrontal cortex, striatum, and hippocampus [4]. The Anterior Cingulate Cortex is associated with cognitive control, impulse control, and attention regulation, and may play a role in regulating and controlling behavior in IA [5]. The amygdala is involved in emotional regulation and reward processing, and may play a role in emotional factors and reinforcement mechanisms in IA [6]. Symptoms of IA may include loss of control over internet use, preoccupation with online activities, neglect of personal responsibilities, and withdrawal symptoms when internet use is reduced or eliminated [7]. A range of negative consequences are associated with IA, including impaired social functioning, academic and work-related problems, and physical and mental health problems. Research has shown that IA is associated with symptoms of ADHD and depressive disorders [8].

In 2013, Internet gaming addiction (IGD) was first introduced by the American Psychiatric Association in the fifth edition of the Diagnostic and Statistical Manual of Mental Disorders (DSM-5) and nine diagnostic criteria for IGD were listed [9]. Although both IA and IGD are related to internet usage, IGD is a specific form of IA, which is addiction to internet games. Therefore, IGD is a subcategory of IA.

While cognitive-behavioral therapy (CBT), medication, and group therapy are common treatments for IA, it is true that there is a lack of data on their effectiveness. This is because research on IA treatment is still relatively new and ongoing. More research is needed to fully understand the most effective treatments for IA and how to tailor treatment approaches to individual needs. Research on IA treatment can help us determine which interventions are most effective for individuals with IA and improve prognosis. In the meantime, clinicians may use a combination of different treatments and strategies to help individuals with IA manage their symptoms and improve their quality of life. This article summarizes the effects of different intervention models on IA and explores the relationship between intervention models and effects.

This study focuses on randomized controlled trials (RCTs) as the research object. This study employed network meta-analysis methods to compare the effectiveness of different treatment methods for treating IA. This study aims to provide reference and evidence-based medicine data for clinical diagnosis and therapy by evaluating the impacts of various intervention models on IA and examining the relationship between various intervention techniques and treatment outcomes.

## Materials and methods

### Inclusion and exclusion standard

Inclusion criteria: (1) Published studies on randomized controlled trials on IA, regardless of whether allocation concealment and blinding were mentioned in Chinese or English language; (2) Study subjects meeting one of two criteria: (a) Diagnosis criteria for IGD in DSM-5, Young's Diagnostic Questionnaire for IA, 1997 American Psychological Association Diagnostic Criteria for IA, or other clinical diagnostic criteria for IA; (b) A score of 40 or higher on either the Internet Addiction Test (IAT) or Chen's Internet Addiction Scale (CIAS) [10–12]; (3) Various scores from relevant internet addiction scales were used as evaluation indicators for the treatment effects of different intervention measures and their combinations; (4) Data extraction was limited to studies with full texts only.

Exclusion criteria: (1) Review systematic reviews; (2) Duplicate literature and non-peer-reviewed material; (3) Studies with outcome indicators that failed to meet the inclusion requirements or had apparent errors or omissions.

### Search strategy

To identify studies that comply with the inclusion criteria, computer searches were performed in several databases, including PubMed, Embase, Web of Science, Wanfang, and China’s National Knowledge Infrastructure (CNKI). The databases were searched up to December 31, 2022, using a combination of controlled vocabulary and free text terms according to each database's search rules. The search terms included: Internet gaming addiction, electronic gaming addiction, online gaming, pathological internet use, addictive internet use, gaming disorder, internet addiction, excessive internet use, computer game addiction, internet dependence, efficacy, randomized, drug therapy, psychotherapy, antidepressants, cognitive behavior, randomized controlled, case–control, clinical trial, intervention, bupropion, methylphenidate, aripiprazole, sertraline, and fluoxetine. Two investigators independently conducted computer and manual searches, with a third expert consulted in the event of disagreement.

### Data extraction

Two researchers independently conducted literature screening following the predetermined inclusion and exclusion criteria. The retrieved literature was imported into literature management software and initially screened by title and abstract after duplicate removal. This led to a thorough reading of the complete text and the final decision regarding inclusion. All data were cross-checked and extracted from the included literature and any discrepancies were resolved by consultation with a third party. The data extracted included basic information about the literature, intervention measures, and outcome indicators.

### Quality assessment

Two researchers strictly assessed the quality of the included studies according to the Cochrane Handbook for Systematic Reviews of Randomized Trials. Cochrane Handbook for Systematic Reviews of Interventions Version 5.1.0. The Cochrane Collaboration). The tool includes seven items: random sequence generation, allocation concealment, blinding of participants and personnel, blinding of outcome assessment, incomplete outcome data, selective reporting, and other biases. Each item was assessed as "low risk," "unclear risk," and "high risk."

### Statistical analysis

The network Meta-analysis package of Stata14.2 was used for network meta-analysis and drawing the network map, and RevMan5.4 was used to evaluate the quality of the included studies. In the statistical process of network meta-analysis, since the outcome measurement indicators of the included studies were continuous data and the scale scoring methods of each study were different, the standard mean difference (SMDs) of different studies and the corresponding 95% confidence intervals (CIs) were used as the effect size to merge the results. First of all, the collected data is tested with the inconsistency model to check whether there is good consistency between the groups and the local areas. If there is consistency, the consistency model is used for further analysis of the data; if the inconsistency is significant, the consistency model cannot be used for subsequent steps. Then, the processed data were sorted using surface under the cumulative ranking curve (SUCRA) values [13], and all the processing results were summarized in a rank-heat map [14] to obtain the ranking of the therapeutic effects of various interventions. The SUCRA value refers to the area under the cumulative sequencing probability curve of an intervention, with the value ranging from 0 to 100%. League tables were also drawn to analyze pairwise comparisons between interventions, including SMD values and 95% confidence intervals. Finally, the funnel plot was drawn using Stata 14.2 to identify whether there was a small sample effect.

## Results

### Literature search

**Fig. 1:**
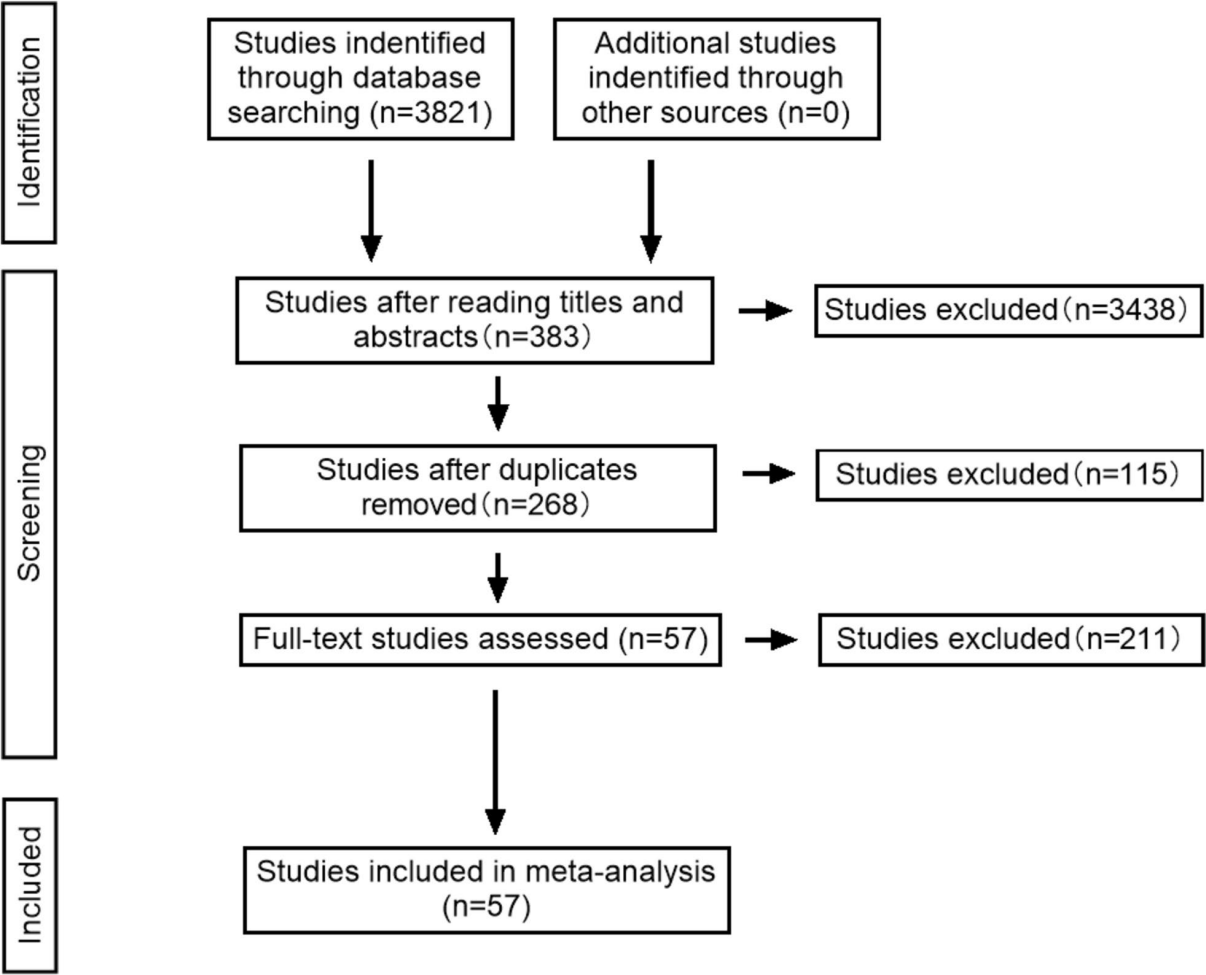
Steps of literature retrieval

A preliminary search yielded 809 English-language studies and 3012 Chinese-language studies. After reviewing the titles and abstracts, 383 articles remained; following a duplication check and full-text reading, 57 studies were finally included for the network meta-analysis, comprising 63 comparisons. The steps for literature retrieval are shown in Fig. 1.

### Characteristics of the included studies

The characteristics of 57 included studies [3, 15–70] are shown in Table 1, which involved 13 interventions, including psychotherapy/CBT, group psychotherapy, Mindfulness-Based Cognitive Therapy (MBCT), placebo/non-intervention, health education, exercise therapy, electro-acupuncture, drug, EEG biofeedback, rTMS, rTMS + CBT, drug + others, and electro-acupuncture + CBT. A total of 3538 patients with IA were included, and all included studies had comparable descriptions.

**Table 1:** Description of included studies

First author	Year	Gender	Age		Comparison	Sample size	Scale	Treatment time
Yuan JJ	2022	male:8, female:6/male:9, female:5		14.28 ± 1.73/13.92 ± 1.89	MBCT/Health education	14/14	IAT	7d
Lu ZJ	2021	male:7, female:10/male:7, female:9		not mentioned	Placebo(non-intervention)/Group psychotherapy	17/16	IAT	5w
Zhong YH	2020	male:19, female:13/male:22, female:10		20.23 ± 4.50/21.62 ± 3.72	rTMS/Placebo(non-intervention)	32/32	IAT	4w
	2020	male:19, female:13/male:22, female:10		20.23 ± 4.50/21.62 ± 3.72	rTMS/Placebo(non-intervention)	32/32	VAS	4w
Zhang LB	2018	male:20, female:13/male:19, female:14		17.15 ± 2.00/17.18 ± 1.78	Psychotherapy (CBT)/Health education	30/30	IAT	12w
Cheng GJS	2017	male:21, female:4/male:23, female:2		15.37 ± 2.31/16.03 ± 1.72	EEG biofeedback/Group psychotherapy	25/25	IAT	12w
Yu YG	2017	not mentioned		not mentioned	Exercise therapy/Placebo(non-intervention)	26/26	IAT	8w
Li M	2014	not mentioned		15.41 ± 1.47/15.62 ± 1.78	Exercise therapy/Placebo(non-intervention)	27/24	IAT	10w
Zhang CS	2013	not mentioned		not mentioned	Exercise therapy/Placebo(non-intervention)	30/30	IAT	16w
Yang Y	2013	not mentioned		20.13 ± 1.77/21.65 ± 2.36	Electro-acupuncture/Group psychotherapy	15/14	IAT	6w
Wang QZ	2012	not mentioned		not mentioned	Psychotherapy (CBT)/Health education	36/36	IAT	12w
Zhu TM	2011	male:24, female:15/male:24, female:1/male:25, female:12		21.00 ± 1.97/22.53 ± 2.26/ ± 22.41 ± 2.10	Electro-acupuncture/Psychotherapy(CBT)/ Electro-acupuncture + CBT	39/36/37	IAT	3w
	2011	male:24, female:15/male:24, female:1/male:25, female:12		21.00 ± 1.97/22.53 ± 2.26/ ± 22.41 ± 2.10	Electro-acupuncture/Psychotherapy(CBT)/ Electro-acupuncture + CBT	39/36/37	VAS	3w
Zheng WF	2007	not mentioned		not mentioned	Group psychotherapy/Placebo(non-intervention)	30/31	IAT	8w
Chen J	2022	not mentioned		not mentioned	rTMS + CBT/Psychotherapy(CBT)	23/22	IAT	8w
	2022	not mentioned		not mentioned	rTMS + CBT/Psychotherapy(CBT)	23/22	VAS	8w
Peng WX	2015	not mentioned		not mentioned	Group psychotherapy/Placebo(non-intervention)	28/28	IAT	8w
Gao J	2012	not mentioned		not mentioned	Exercise therapy/Placebo(non-intervention)	35/34	IAT	12w
Yao LH	2012	not mentioned		not mentioned	Group psychotherapy/Placebo(non-intervention)	19/19	IAT	12w
Duan SL	2012	male:7, female:4/male:7, female:4		not mentioned	Group psychotherapy/Placebo(non-intervention)	11/11	IAT	13w
Zhang W	2022	not mentioned		19.61 ± 1.19/19.39 ± 1.73	Placebo(non-intervention)/Exercise therapy	23/23	IAT	12w
Yang WJ	2022	male:6, female:16/male:7, female:14		19.9 ± 0.8/19.5 ± 0.8	Placebo(non-intervention)/Group psychotherapy	22/21	IAT	1w
Alavi SS	2021	male:14, female:4/male:17, female:6		21.7 ± 4.2/21.04 ± 3.7	Group psychotherapy/Psychotherapy(CBT)	18/23	IAT	12w
Peng W	2021	male:24, female:12/male:25, female:12/male:24, female:15		22.53 ± 2.26/22.41 ± 2.10/21.00 ± 1.97	Psychotherapy(CBT)/Electro-acupuncture + CBT/Electro-acupuncture	36/37/39	IAT	6w
Yang Y	2017	male:13, female:2/male:13, female:1		21.13 ± 1.30/21.65 ± 2.36	Electro-acupuncture/Group psychotherapy	15/14	IAT	6w
Li H	2017	not mentioned		22.5 ± 2.3/21.0 ± 2.0/22.5 ± 2.0	Psychotherapy(CBT)/Electro-acupuncture/ Electro-acupuncture + CBT	36/39/37	IAT	6w
Sun JJ	2022	not mentioned		17.36 ± 3.98/18.90 ± 4.45	rTMS/Placebo(non-intervention)	31/30	CIAS	2w
	2022	not mentioned		17.36 ± 3.98/18.90 ± 4.45	rTMS/Placebo(non-intervention)	31/30	VAS	2w
Chen D	2021	male:24, female:12/male:26, female:12		16.89 ± 1.43/16.52 ± 1.26	Health education/Group psychotherapy	36/38	CIAS	8w
Song GY	2020	not mentioned		not mentioned	Placebo(non-intervention)/Group psychotherapy	60/60	CIAS	20w
Li R	2020	not mentioned		not mentioned	Psychotherapy(CBT)/Group psychotherapy	12/12	CIAS	8w
Chen AW	2020	male:35, female:10/male:36, female:9		14.83 ± 2.46/14.95 ± 2.51	Health education/Group psychotherapy	45/45	CIAS-R	8w
Xu ZX	2018	male:16, female:4/male:14, female:6		13.3 ± 1.3/14.0 ± 1.0	Health education/Group psychotherapy	20/20	CIAS	4w
Chen SZ	2018	male:34, female:18/male:36, female:16		16.01 ± 1.45/16.15 ± 1.42	Psychotherapy(CBT)/Group psychotherapy	52/52	CIAS	/
Tang RZH	2018	male:18, female:6/male:23, female:4/male:22, female:4		22.63 ± 5.23/22.04 ± 7.54/21.54 ± 5.77	Electro-acupuncture/Psychotherapy(CBT)/ Electro-acupuncture + CBT	24/27/26	CIAS-R	8w
	2018	male:18, female:6/male:23, female:4/male:22, female:4		22.63 ± 5.23/22.04 ± 7.54/21.54 ± 5.77	Electro-acupuncture/Psychotherapy (CBT)/Electro-acupuncture + CBT	24/27/26	VAS	8w
Zhao ST	2016	male:59, female:6/male:58, female:7		16.2 ± 4.1/16.5 ± 3.6	Health education/Group psychotherapy	65/65	CIAS	16w
Zhou M	2015	male:7, female:11/male:8, female:10		20.88 ± 1.13/20.38 ± 1.41	Group psychotherapy/Placebo(non-intervention)	18/18	CIAS-R	8w
Wu ZX	2015	not mentioned		not mentioned	Group psychotherapy/Placebo(non-intervention)	15/15	CIAS	8w
Ming LJ	2014	not mentioned		not mentioned	Psychotherapy (CBT)/Placebo(non-intervention)	60/60	CIAS	10w
Zhang XF	2013	not mentioned		19.83 ± 1.07/20.1 ± 1.07	Health education/MBCT	42/42	CIAS	1w
Lv WQ	2012	not mentioned		not mentioned	Group psychotherapy/Placebo(non-intervention)	26/26	CIAS	6w
Chen ZZ	2011	not mentioned		not mentioned	Group psychotherapy/Placebo(non-intervention)	14/23	CIAS-R	7w
Li G	2009	male:35, female:3/male:33, female:5		17 ± 4/16 ± 4	Psychotherapy(CBT)/Placebo(non-intervention)	38/38	CIAS	8-10w
Wang GR	2008	not mentioned		not mentioned	Group psychotherapy/Placebo(non-intervention)	24/24	CIAS	12w
Cao FL	2007	not mentioned		not mentioned	Group psychotherapy/Health education	26/31	CIAS	8w
Bai Y	2007	male:20, female:4/male:20, female:4		19 ± 2/19 ± 2	Group psychotherapy/Placebo(non-intervention)	24/24	CIAS	4w
Zhao Y	2022	male:35, female:15/male:33, female:17		15.16 ± 2.18/15.25 ± 2.12	Health education/Group psychotherapy	50/50	CIAS-R	12w
Liu X	2021	male:47, female:14/male:44, female:16		15.3 ± 0.8/15.2 ± 0.8	Health education/MBCT	61/60	CIAS	8w
Deng LY	2017	not mentioned		21.86 ± 1.90/22.05 ± 1.81	Group psychotherapy/Placebo(non-intervention)	44/19	CIAS	6w
	2017	not mentioned		21.86 ± 1.90/22.05 ± 1.81	Group psychotherapy/Placebo(non-intervention)	44/19	VAS	6w
Liu D	2013	not mentioned		20.3 ± 0.9/20.3 ± 1.0	Group psychotherapy/Health education	16/15	IAT	12w
Tang RZH	2017	not mentioned		not mentioned	Psychotherapy(CBT)/Electro-acupuncture/ Electro-acupuncture + CBT	20/23/21	VAS	4w
Zhang Y	2016	male:25, female:9/male:27, female:7		17.21 ± 2.12/16.44 ± 3.53	Electro-acupuncture/Placebo(non-intervention)	34/34	VAS	6w
Zhang HL	2011	not mentioned		not mentioned	Exercise therapy/Placebo(non-intervention)	18/18	IAT	12w
Deng YQ	2014	not mentioned		not mentioned	Exercise therapy/Placebo(non-intervention)	24/24	CIAS	10w
Fu YS	2016	not mentioned		not mentioned	Exercise therapy/Placebo(non-intervention)	42/42	IAT	16w
Yang CY	2017	male:12, female:14/male:13, female:13		19.6 ± 1.2/19.7 ± 1.4	Exercise therapy/Placebo(non-intervention)	26/26	CIAS	16w
Ji W	2017	not mentioned		not mentioned	Exercise therapy/Health education	10/10	IAT	12w
Qiu JM	2022	male:20, female:20/male:20, female:20		15.64 ± 1.68/15.59 ± 1.71	Drug/Drug + others	40/40	CIAS-R	8w
Zhou XH	2016	male:28, female:4/male:29, female:3		not mentioned	Drug + others/Health education	32/32	CIAS-R	8w
Song HQ	2017	not mentioned		13.90 ± 1.37/14.08 ± 1.49	Drug/Drug + others	20/20	IAT	16w
Wang DP	2014	not mentioned		not mentioned	Psychotherapy (CBT)/Drug + others	38/36	CIAS	8w

### Risk of bias assessment (Fig. 2)

**Fig. 2:**
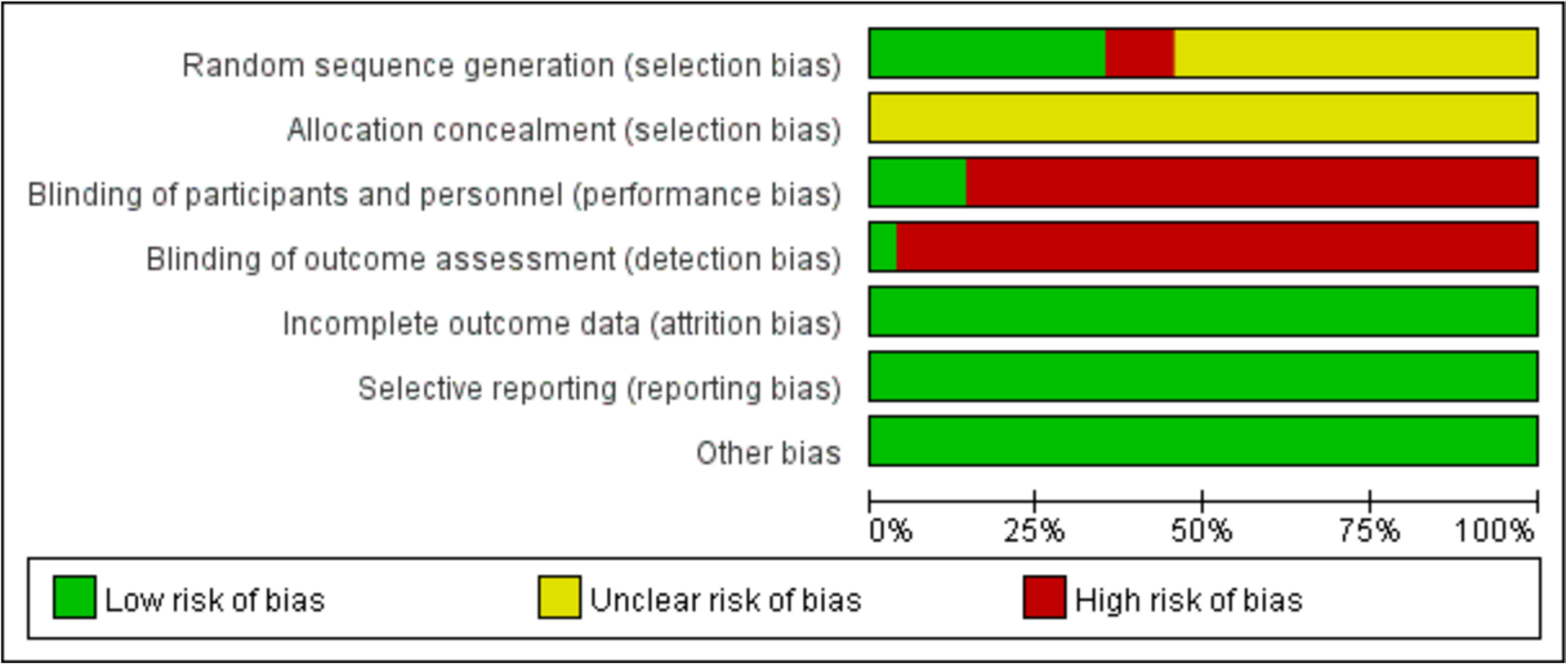
Bias risk assessment for included studies

Two reviewers strictly followed the recommended bias risk assessment tools in the Cochrane Handbook to assess the risk of bias for the included studies. For example, in terms of randomization methods, 22 studies were evaluated as "low risk" as they adopted randomized allocation methods such as random number tables, stratified randomization, and drawing lots. Another 28 studies only mentioned "randomization" without reporting specific randomization methods and were evaluated as "unclear risk." The remaining seven studies randomly assigned patients according to the admission order and were rated "high risk." All studies failed to report whether allocation concealment was performed and was rated as “unclear risk." Seven studies involved blinding and were rated as "low risk." All studies had complete data and were rated as "low risk." Other biases were not mentioned and were rated as "low risk."

### Network meta-analysis

#### Network figure

**Fig. 3:**
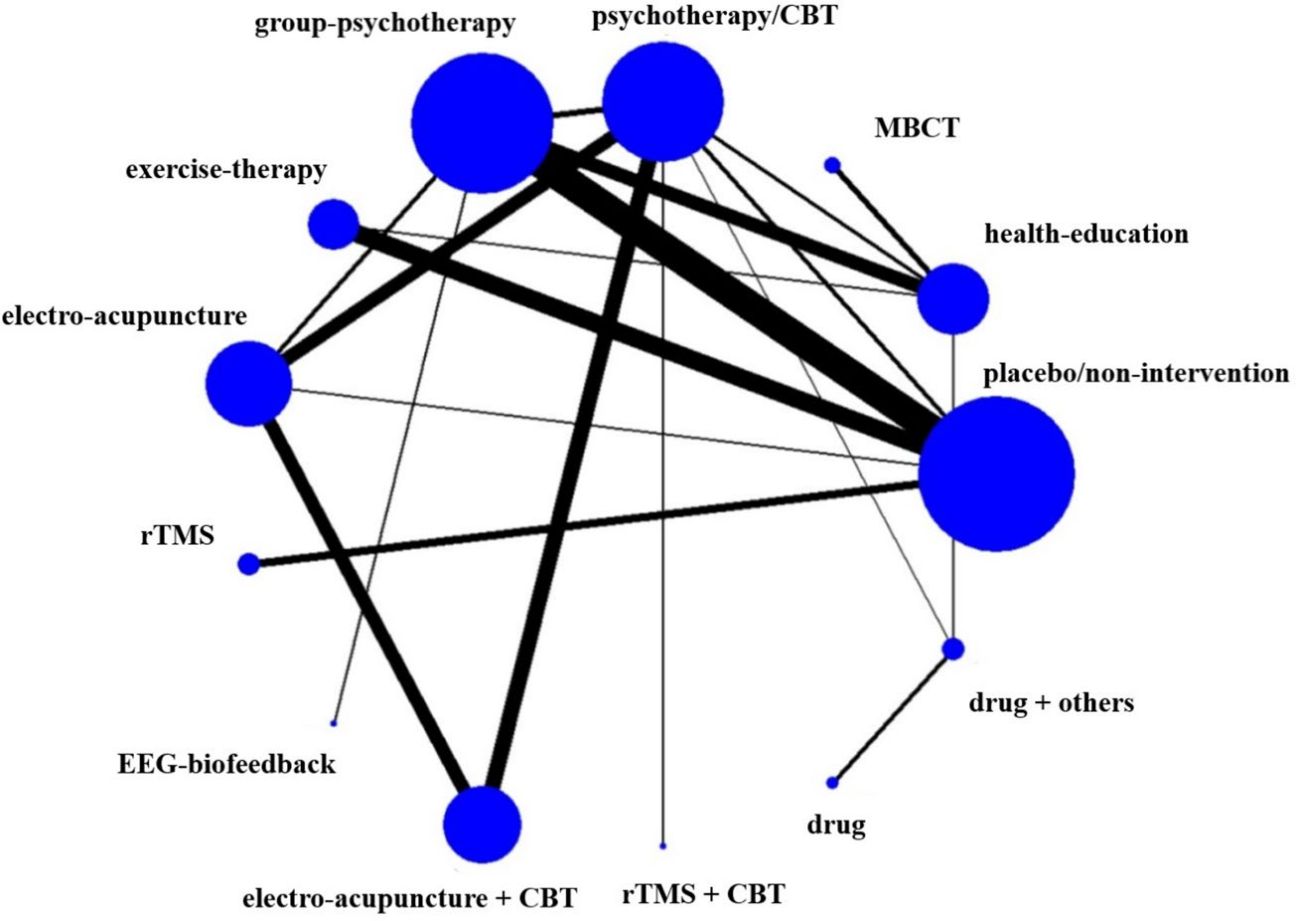
Network figure about efficient evidence

A total of 57 randomized controlled trials (RCTs) reported the effectiveness of different interventions to treat IA, involving 13 interventions. The size of each node in the network diagram (Fig. 3) represents the sample size of the corresponding intervention, and the thickness of the lines that connect different interventions represents the number of studies comparing the two interventions.

#### Analysis result

Network meta-analysis was performed on the included studies, generating 78 pairwise comparisons with 95% confidence intervals for the SMD. Please refer to Table 2 for detailed information.

**Table 2:** Network meta-analysis [SMD (95% CI)]

**Interventions**	Drug + others	Drug	rTMS + CBT	Electro-acupuncture + CBT	EEG biofeedback	rTMS	Electro-acupuncture	Exercise therapy	Group psychotherapy	Psychotherapy/CBT	MBCT	Health education
drug	-2.12 (-3.53,-0.70)											
rTMS + CBT	2.92 (-0.44,6.29)	5.04 (1.39,8.69)										
electro-acupuncture + CBT	-1.01 (-3.64,1.62)	1.11 (-1.88,4.09)	-3.93 (-6.28,-1.58)									
EEG biofeedback	-1.35 (-4.86,2.16)	0.77 (-3.01,4.55)	-4.27 (-7.58,-0.96)	-0.34 (-2.89,2.21)								
rTMS	-0.89 (-3.92,2.14)	1.22 (-2.12,4.57)	-3.82 (-6.61,-1.02)	0.12 (-1.72,1.96)	0.46 (-1.81,2.72)							
electro-acupuncture	-3.42 (-6.87,0.04)	-1.30 (-5.03,2.43)	-6.34 (-9.59,-3.09)	-2.41 (-4.89,0.07)	-2.07 (-4.88,0.74)	-2.52 (-4.71,-0.34)						
exercise therapy	-1.54 (-4.47,1.40)	0.58 (-2.68,3.84)	-4.46 (-7.15,-1.77)	-0.53 (-2.21,1.15)	-0.19 (-2.33,1.95)	-0.65 (-1.84,0.55)	1.88 (-0.17,3.93)					
group psychotherapy	-1.93 (-4.84,0.97)	0.19 (-3.05,3.42)	-4.85 (-7.51,-2.19)	-0.92 (-2.55,0.71)	-0.58 (-2.55,1.38)	-1.04 (-2.16,0.09)	1.49 (-0.52,3.50)	-0.39 (-1.23,0.45)				
psychotherapy/CBT	-1.85 (-4.37,0.67)	0.27 (-2.62,3.16)	-4.77 (-7.00,-2.54)	-0.84 (-1.58,-0.10)	-0.50 (-2.94,1.94)	-0.96 (-2.64,0.73)	1.57 (-0.80,3.94)	-0.31 (-1.82,1.20)	0.08 (-1.37,1.54)			
MBCT	-2.36 (-4.74,0.03)	-0.24 (-3.01,2.53)	-5.28 (-8.16,-2.40)	-1.35 (-3.32,0.62)	-1.01 (-4.06,2.04)	-1.46 (-3.95,1.02)	1.06 (-1.93,4.05)	-0.82 (-3.19,1.55)	-0.43 (-2.76,1.91)	-0.51 (-2.33,1.32)		
health education	-4.25 (-6.34,-2.17)	-2.14 (-4.66,0.39)	-7.17 (-9.82,-4.53)	-3.24 (-4.84,-1.65)	-2.90 (-5.73,-0.08)	-3.36 (-5.56,-1.16)	-0.84 (-3.59,1.92)	-2.71 (-4.78,-0.64)	-2.32 (-4.35,-0.30)	-2.40 (-3.82,-0.99)	-1.90 (-3.05,-0.74)	
placebo/non-intervention	-2.26 (-3.26,-1.26)	0.26 (-1.68,2.20)	-2.05 (-4.52,0.41)	-1.83 (-4.31,0.65)	-1.62 (-2.28,-0.95)	-1.22 (-1.74,-0.71)	-1.16 (-3.02,0.69)	-1.31 (-2.67,0.05)	-0.80 (-3.07,1.48)	1.10 (-0.86,3.06)	-1.03 (-4.03,1.96)	0.84 (-1.33,3.02)

The results of the network meta-analysis showed that compared with the placebo/non-intervention group, drug + others (SMD = -2.26, 95% CI = -3.26 ~ -1.26), EEG biofeedback (SMD = -1.62, 95% CI = -2.28 ~ -0.95), rTMS (SMD = -1.22, 95% CI = -1.74 ~ -0.71) showed statistical significance in the treatment effect of IA. Compared to the health education group, drug + others (SMD = -4.25, 95% CI =—6.34 ~ -2.17), rTMS + CBT (SMD = -7.17, 95% CI = -9.82 ~ -4.53), electro-acupuncture + CBT (SMD = -3.24, 95% CI = -4.84 ~ -1.65), EEG biofeedback (SMD = -2.90, 95% CI = -5.73 ~ -0.08), rTMS (SMD = -3.36, 95% CI = -5.56 ~ -1.16), exercise therapy (SMD = -2.71, 95% CI = -4.78 ~ -0.64), group psychotherapy (SMD = -2.32, 95% CI = -4.35 ~ -0.30), psychotherapy/CBT (SMD = -2.40, 95% CI = -3.82 ~ -0.99), and MBCT (SMD = -1.90, 95% CI = -3.05 ~ -0.74) have been shown to be statistically significant in the treatment of IA. Compared to the psychotherapy/CBT group, rTMS + CBT (SMD = -4.77, 95% CI = -7.00 ~ -2.54), electro-acupuncture + CBT (SMD = -0.84, 95% CI = -1.58 ~ -0.10), reflect the difference in therapeutic effect compared to use CBT alone, combined physical therapy is essential for the curative effect. Similarly, drug + others (SMD = -2.12, 95% CI = -3.53 ~ -0.70) also showed statistically different advantages compared to interventions that only used drugs. Furthermore, compared to MBCT, psychotherapy/CBT group, psychotherapy, exercise therapy, electroacupuncture, rTMS, EEG biofeedback, and electroacupuncture + CBT, therapeutic efficacy in the rTMS + CBT group showed optimal differences, and the results were statistically significant (*p* < 0.05), which further demonstrating the unique therapeutic effect of rTMS + CBT. This combination of treatment modalities could provide a reference for the treatment of IA in the future.

#### Rank

**Fig. 4:**
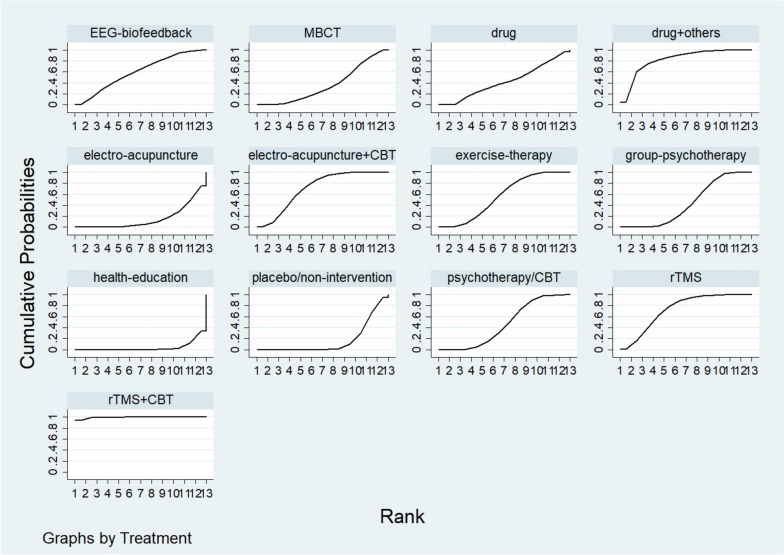
Cumulative probabilities

In terms of efficiency, the ranking was rTMS + CBT > drug + others > rTMS > electro-acupuncture + CBT > EEG-biofeedback > exercise-therapy > psychotherapy/CBT > group-psychotherapy > drug > MBCT > placebo/non-intervention > electro-acupuncture > health-education. The specific rank order is shown in Table 3, and the cumulative probabilities are shown in Fig. 4.

**Table 3:** The ordering results of network meta-analysis

ID and rank	Treatment												
	placebo/non-intervention	health-education	MBCT	psychotherapy/CBT	group-psychotherapy	exercise-therapy	electro-acupuncture	rTMS	EEG-biofeedback	electro-acupuncture + CBT	rTMS + CBT	drug	drug + others
Best	0.0	0.0	0.0	0.0	0.0	0.0	0.0	0.2	0.4	0.0	94.6	0.0	4.8
2nd	0.0	0.0	0.3	0.0	0.0	1.2	0.1	15.7	12.5	9.0	4.6	0.5	56.1
3rd	0.0	0.0	2.4	0.5	0.5	5.1	0.3	24.4	15.0	22.8	0.6	13.8	14.5
4th	0.0	0.0	4.7	4.3	2.5	12.4	0.4	22.3	12.7	24.5	0.1	8.9	7.2
5th	0.0	0.0	6.0	10.0	6.6	18.3	0.9	16.8	10.7	18.1	0.1	7.5	5.0
6th	0.0	0.0	7.5	15.8	13.4	20.0	1.6	10.0	9.4	11.8	0.0	6.6	3.8
7th	0.3	0.0	8.6	19.4	18.9	17.8	2.3	5.6	9.5	8.3	0.1	5.8	3.3
8th	1.6	0.3	10.6	22.9	22.7	12.7	4.4	2.9	8.5	3.8	0.0	7.1	2.6
9th	8.1	0.7	15.1	16.5	20.4	8.2	7.9	1.4	7.9	1.5	0.0	10.8	1.6
10th	19.1	1.9	20.2	8.8	12.7	3.9	10.9	0.6	7.9	0.3	0.0	13.2	0.8
11th	39.1	9.6	13.9	1.6	2.3	0.4	19.4	0.1	3.4	0.0	0.0	10.1	0.2
12th	26.9	21.7	10.7	0.3	0.1	0.0	26.7	0.0	1.5	0.0	0.0	12.1	0.0
Worst	4.8	65.9	0.0	0.1	0.0	0.0	25.2	0.0	0.6	0.0	0.0	3.5	0.0

### Inconsistency test

The consistency of each closed-loop result was tested. Inconsistency factors (IF) showed *p* = 0.4042, indicating good consistency. All local *p* > 0.05, indicating good consistency among all groups.

### Publication bias

**Fig. 5:**
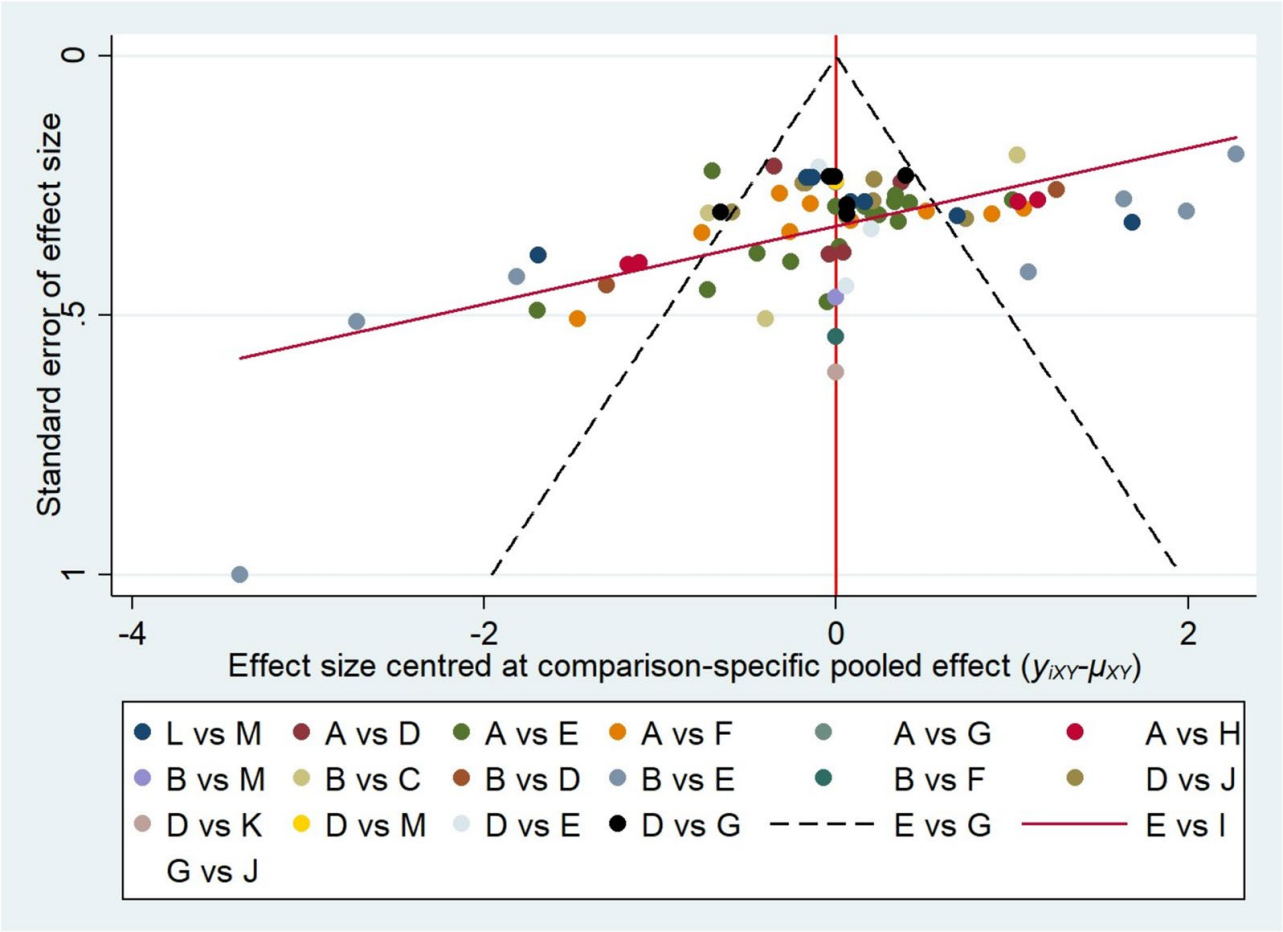
Funnel plot about the 14 interventions in the treatment of IA. **A** placebo/non-intervention, **B** health-education, **C** MBCT, **D** psychotherapy/CBT, **E** group-psychotherapy, **F** exercise-therapy, **G** electro-acupuncture, **H** rTMS, **I** EEG-biofeedback, **J** electro-acupuncture + CBT, **K** rTMS + CBT, **L** drug, **M** drug + others

The research was roughly symmetrically distributed on both sides of the midline, indicating that a small sample effect was less likely to exist as shown in Fig. 5.

## Discussion

IA causes serious physical and mental distress and potential harm to people. Many scholars have investigated prevention and intervention measures for IA in recent years, resulting in various interventions. There have been relatively few studies on treating IA, and the effects of most treatments are limited. To investigate the efficacy, advantages, and disadvantages of different treatment methods used alone or in combination, this study conducted a network meta-analysis of the efficacy of 13 intervention methods. The ranking results showed that the top four intervention measures in terms of effectiveness were rTMS combined with CBT, drug combination with other treatments, rTMS, and electro-acupuncture combined with CBT. The rankings of various comprehensive treatments were also high, indicating that combined therapy can effectively improve the effect of IA compared to using a single intervention measure for treatment.

### rTMS combined with CBT

Based on the efficacy ranking, both rTMS combined with CBT and rTMS alone achieved optimal treatment effects. Compared with all other interventions except for drug-combined comprehensive treatment, rTMS combined with CBT has shown statistical differences. rTMS is a physical therapy that generates a sequence of repetitive electromagnetic pulses through an electromagnetic coil. It can regulate cortical excitability by acting on specific cortical regions of the brain [71]. Numerous domestic and foreign studies have shown that rTMS has immense potential to treat substance dependence [72]. Studies have shown that rTMS treatment targeting the left or right dorsolateral prefrontal cortex (DLPFC) can fully mobilize the cognitive regulatory capacity of the DLPFC, increase the excitability of cortical areas, and regulate activity by maintaining functional levels of dopamine and other neurotransmitters in various structures of the reward circuit [73]. Thus, people's cravings for the Internet will be reduced, and their addictive behavior will be curtailed. Furthermore, neuroimaging studies have also found that rTMS applied to DLPFC can effectively inhibit brain cortices related to addictive behavior and sensation, reduce the craving of participants with IA, improve their cognitive control and emotional regulation abilities, reward and cognitive control systems, and thereby reduce the craving and behavior in IA [74, 75]. IA can significantly reduce the white matter integrity of DLPFC compared to normal individuals [76], similar to the changes observed in drug addiction. Excessive use of the internet can alter the reward and pleasure centers of the brain, making it difficult to quit addiction. rTMS can help regulate brain activity and reduce cravings related to IA.

Simultaneously, CBT is also a major intervention method for IA in psychotherapy, which is effective in many randomized controlled studies on IA [77, 78]. The essence of CBT is to correct the cognitive dysfunction of patients with IA. Improving cognitive control ability may be the key to solving IA [79]. The results of the network meta-analysis demonstrated that rTMS combined with CBT treatment was more effective than rTMS or CBT alone. When people try to reduce or withdraw from online activities, IA can lead to withdrawal symptoms. Physical therapy interventions can help control these physiological and emotional withdrawal symptoms, making it easier for patients to receive psychological treatment. Therefore, people's desire for the internet will decrease and their addictive behavior will be restricted [80]. This could be attributed to the potential of combined treatment to simultaneously improve the physiological, psychological, and behavioral aspects of IA, thereby producing a cumulative effect for better outcomes.

### Combination of medication and other therapies

Medication combined with other intervention measures, as a comprehensive intervention measure, demonstrates excellent efficacy, outperforming single medication therapy. Many experts believe that IA indicates impulse control disorders over the internet. This addictive behavior is classified as compulsive behavior [81]. Researchers suggest that selective serotonin reuptake inhibitors (SSRIs), as first-line drugs for treating obsessive–compulsive disorder, may also have optimal therapeutic effects on patients with IA. The results of our meta-analysis indicated that the efficacy of single drug therapy is only superior to that of the general intervention group, the placebo group, and the single electro-acupuncture group and is comparable to that of a single psychological treatment. However, when combined with psychological or physical treatment, it demonstrates excellent results and is statistically significant compared to single-medication therapy. This may result from medication therapy controlling anxiety and depression to a certain extent in IA patients. For IA patients with poor self-control and resistance to treatment, applying medication first to stabilize the patient's emotions and then using psychological or physical treatment can further improve their depressive and anxious symptoms and cognitive function, thereby reducing their craving for the internet. Additionally, combined therapy can reduce the adverse effects of the long-term use of single medications and improve treatment safety, resulting in better outcomes.

### Electro-acupuncture and CBT combined treatment

We also noticed that the combination of electro-acupuncture and CBT treatment had achieved unexpected results. Traditional Chinese medicine has a long history of application in mental illnesses, such as using electro-acupuncture to treat depression and sleep disorders. Studies have demonstrated that electro-acupuncture can promote the recovery of neurons in the affected brain area [82]. The acupoints used in acupuncture can increase blood flow or induce electrical potentials in specific brain regions [83]. As a commonly used treatment in traditional Chinese medicine, Electro-acupuncture therapy has gained recognition in treating modern addictive behaviors. The combination of electro-acupuncture and CBT treatment was shown to significantly reduce the addiction level and related clinical symptoms of IGD patients. fMRI is increasingly being used to study the mechanism of acupuncture. Previous studies have confirmed that acupuncture and moxibustion can regulate the structure and function of the brain regions of drug addicts and can regulate the functional connection between the reward and habit systems of IA [84]. Therefore, the regulatory effect of acupuncture on the brain area of network addiction patients may be a potential mechanism of acupuncture in-network addiction. Both electro-acupuncture and cognitive-behavioral therapy have significant positive effects on network-addicted adolescents. Both treatment methods effectively improve network addiction patients' psychological experience and behavioral expression. However, electro-acupuncture is more effective than psychological therapy regarding impulse control and neuron protection. This advantage may be related to increased NAA and CHO levels in the prefrontal cortex and anterior cingulate cortex [82]. The combination of electro-acupuncture and psychological interventions (cognitive-behavioral therapy, group, psychological therapy combined with individual psychological therapy) can alleviate the mental symptoms, sleep quality, and impulse characteristics of network addiction patients [85]. Its mechanism may be related to increased brain sensory perception gate function. Psychological intervention can help relieve the fear of electro-acupuncture and improve the treatment effect, as acupuncture is an exogenous stimulation that usually accompanies pain. Combining CBT with electro-acupuncture treatment has shown superior results compared to using acupuncture alone, suggesting a synergistic effect between electro-acupuncture and CBT. In summary, combining electro-acupuncture and CBT can achieve optimal therapeutic outcomes.

### Other interventions

Compared to comprehensive treatment, we have observed that the following five interventions, namely health education, electro-acupuncture, place/non-intervention, MBCT, and drug treatment, have relatively lower effectiveness. We believe this may be attributed to the following reasons. Health education often focuses on encouragement, comfort, and providing knowledge related to IA, but it fails to address the deep-rooted inner struggles of patients and alleviate their dependency on the internet. Therefore, health education is generally less effective in most cases. As for electroacupuncture treatment, studies have shown that IA patients often experience strong withdrawal reactions during the initial stages of treatment, such as restlessness, palpitations, and irritability. As an acupuncture treatment, electroacupuncture may cause swelling and heaviness at the treatment site, and discomfort may persist for a period of time after the needles are removed and gradually subside. Consequently, the compliance and expectations of most patients are not high, resulting in mediocre therapeutic effects of using electroacupuncture alone.

MBCT, as a mindfulness and meditation-oriented treatment method, has been shown in research to reduce craving for IA by improving the understanding ability of IA patients, improving loneliness in IA, and reducing heart rate and cortisol levels [23]. However, due to the impulsive personality traits of most addiction patients, many individuals are unable to calm their minds and fully engage in the understanding and experience provided by MBCT therapy. Therefore, relying solely on MBCT treatment is ineffective.

The use of drugs alone also fails to achieve better therapeutic effects, mainly because in simple applications of drug therapy, common SSRIs such as sertraline, which selectively inhibit the reuptake of serotonin by central neurons, leading to an increase in serotonin concentration, have slow-acting effects and are associated with side effects such as gastrointestinal discomfort. Consequently, patients have low long-term medication tolerance and poor treatment effectiveness. Moreover, the cognitive function of patients is still insufficient in medication-only treatment [3]. On the other hand, combination therapy and comprehensive treatment can reduce the adverse reactions caused by long-term medication use, enhance treatment safety, and improve treatment effectiveness. Therefore, a single treatment approach may not be able to address all the issues, and personalized comprehensive treatment plans should be developed based on the unique characteristics of each patient during clinical treatment to ensure compliance and treatment effectiveness.

IA is a complex multifactorial disorder with numerous physiological, psychological, and social elements. A single treatment plan can have difficulty addressing all issues, while a comprehensive therapy can integrate the advantages and disadvantages of various treatments and take targeted measures. Although the findings of this study confirm the safety and feasibility of electro-acupuncture + CBT therapy for IA, there are still some limitations. Due to time constraints, the long-term efficacy of the subjects has not been observed, and the effectiveness assessment was based on clinical scales. Therefore, it is possible that subjects may have concealed some information during the measurement.

### Limitation

(1) Most of the studies were conducted by Chinese researchers. (2) The literature on rTMS combined with CBT treatment and drug combinations with other treatments included in this study was limited.

## Conclusion

The results of our study show that, although all treatments were slightly more effective than placebo/non-intervention and health education in treating IA patients, rTMS + CBT had the best therapeutic effect in treating IA patients with different interventions, followed by drugs combined with other treatments, followed by rTMS and electro-acupuncture + CBT. This proves the unique role physical therapy, specifically rTMS therapy, plays in treating patients with IA. Comprehensive intervention can achieve better therapeutic effects than using drugs or psychotherapy alone by combining drug therapy, physical therapy, and psychotherapy. Comprehensive intervention improves the physical, psychological, and behavioral aspects of patients with IA by combining the benefits of various methods.

## Data Availability

All data generated or analysed during this study are included in this published article.
